# Distribution of Culturable Phosphate-Solubilizing Bacteria in Soil Aggregates and Their Potential for Phosphorus Acquisition

**DOI:** 10.1128/spectrum.00290-22

**Published:** 2022-05-10

**Authors:** Donglan He, Wenjie Wan

**Affiliations:** a College of Life Science, South-Central University for Nationalities, Wuhan, People’s Republic of China; b Wuhan Botanical Garden, Chinese Academy of Sciences, Wuhan, People’s Republic of China; c State Key Laboratory of Agricultural Microbiology, Huazhong Agricultural University, Wuhan, People’s Republic of China; USDA–San Joaquin Valley Agricultural Sciences Center

**Keywords:** colony number, fertilization treatment, gene abundance, halo diameter, phosphorus transformation, plant-growth-promoting ability

## Abstract

Deciphering distribution patterns of phosphate-solubilizing bacteria (PSB) and phosphorus-cycling-related genes in soils is important to evaluate phosphorus (P) transformation. However, the linkage between PSB number and P-cycling-related gene abundance in soils, especially soil aggregates, remains largely unknown. Here, we estimated the numbers of PSB and abundances of P-cycling-related genes (i.e., *gcd* and *bpp*) in soil aggregates under different fertilization regimes as well as P-solubilizing performance and plant-growth-promoting ability of PSB. We found that tricalcium phosphate-solubilizing bacteria, phytate-degrading bacteria, and *gcd* and *bpp* abundances were more abundant in silt plus clay (silt+clay; <53 μm) than in macroaggregate (250 to 2000 μm) and microaggregate (53 to 250 μm). Fertilization treatment and aggregate fractionation showed distinct effects on PSB number and P-cycling-related gene abundance. We found significantly negative correlation between *gcd* gene abundance and tricalcium phosphate-solubilizing bacterial number (Col-CaP) and dramatically positive correlation between *bpp* gene abundance and phytate-degrading bacterial number (Col-Phy). P fractions were responsible for PSB number and P-cycling-related gene abundance. The isolated Pseudomonas sp. strain PSB-2 and *Arthrobacter* sp. strain PSB-5 exhibited good performances for solubilizing tricalcium phosphate. The inoculation of Pseudomonas sp. PSB-2 could significantly enhance plant fresh weight, plant dry weight, and plant height. Our results emphasized distinct distribution characteristics of PSB and P-cycling-related genes in soil aggregates and deciphered a close linkage between PSB number and P-cycling-related gene abundance. Our findings might guide the isolation of PSB from agricultural soils and provide a candidate plant-growth-promoting bacterium for agro-ecosystems.

**IMPORTANCE** Phosphate-solubilizing bacteria are responsible for inorganic P solubilization and organic P mineralization. Elucidating the linkage between phosphate-solubilizing bacterial number and P-cycling-related gene abundance is important to isolate plant-growth-promoting bacteria for agro-ecosystems. Our findings reveal differentiating strategies of phosphate-solubilizing bacteria in soil aggregates, and the deciphered P fractions show strong effects on distribution patterns of phosphate-solubilizing bacteria and P-cycling-related genes. Additionally, we isolated phosphate-solubilizing bacteria with good plant-growth-promoting ability. This study enriches our knowledge of P cycling in soil aggregates and might guide the production and management of farmland.

## INTRODUCTION

Phosphorus limitation is pervasive in both terrestrial and aquatic ecosystems (1, 2) and affects the growth and development of plants (3). Most P in soils presents inorganic insoluble (e.g., tricalcium phosphate) and organic insoluble/soluble forms (e.g., phytate and nucleic acid) (4, 5). Only orthophosphate (H_2_PO_4–_ or HPO_4_^2–^) can directly be absorbed by living plants (6). In agro-ecosystems, the source of soil P is mainly from the application of inorganic P (IP) (e.g., tricalcium phosphate) and organic P (OP)-containing fertilizers (e.g., plant and animal residues) (6–8). However, this externally added P (i.e., IP and OP) can easily get converted into salts and become insoluble by binding to Ca, Al, Fe, Mg, and Mn (4, 9). The transformation of plant-unavailable P to plant-available P requires the solubilization for IP and mineralization for OP by phosphate-solubilizing microorganisms (4, 8–10). Therefore, understanding the content of P fractions and abundance of phosphate-solubilizing bacteria is of great importance to predict and estimate P mobility and turnover as well as to guide rational fertilization.

The solubilization of IP requires organic acid (e.g., acetic acid, oxalic acid, gluconic acid, and lactic acid) released by microorganisms (9, 11). Previous studies have reported that the *gcd* gene, encoding glucose dehydrogenase, can be found in some specific bacteria (e.g., Pseudomonas frederiksbergensis and Acinetobacter pittii) and participate in the oxidation of glucose to gluconic acid (11, 12). In contrast, the mineralization of OP (e.g., phosphoesters and phytate) needs the function of extracellular enzymes (e.g., phosphatase and phytase) mainly produced by microorganisms (13, 14). Phytate, accounting for 80% of total soil OP, is the major compound for OP storage in plants and is extremely stable (5, 15). Phytase (EC 3.1.3.8), alternatively known as *myo*-inositol phosphohydrolases, are produced by phytate-degrading bacteria (14, 15). Earlier studies have reported that the *bpp* gene, encoding β-propeller phytase, is widely distributed in soils (15, 16). Consequently, the *gcd* and *bpp* genes can be good biomarkers to evaluate solubilization for IP and mineralization for OP, respectively.

Earlier studies have reported that tricalcium phosphate-solubilizing bacteria and phytate-degrading bacteria can promote plant growth via enhancing soil availability P (17–20). Recent studies have reported that PSB mainly belongs to Gram-negative bacteria (e.g., Acinetobacter, *Citrobacter*, *Massilia*, and Pseudomonas) (3, 21–24), whereas some are Gram-positive bacteria (e.g., *Bacillus*) (17). For instance, Bacillus subtilis strain KPS-11 can produce indole-3-acetic acid and extracellular phytase, which in turn significantly promotes vegetation properties including height, fresh weight, and dry weight of potato (Solanum tuberosum L.) (17). Consequently, extensive efforts have been made to provide candidate PSB for agro-ecosystems (13, 14, 25, 26). From the practical viewpoint, the application potentials of soil-derived PSB for agro-ecosystems would be better when considering biosafety and bacterial adaptability to the environment. Therefore, disentangling distribution patterns of PSB in agricultural soils is useful to guide isolation of PSB.

Soil aggregates regarded as microenvironments can determine nutrient distribution and gas (e.g., oxygen and carbon dioxide) circulation (8, 27) and therefore affect microbial distribution (8, 28). Studies involved in phosphorus cycling are mainly about phosphorus fractionation in environments and gene abundance of phosphate-solubilizing bacteria in different habitats (e.g., soils, sediments, and water), as well as PSB isolation (3–9, 13–17). However, a limited study has reported culturable phosphate-solubilizing bacterial abundance in soil aggregates with different fertilization treatments. Fertilization treatment and aggregate fractionation affect P fractionation (8); therefore, knowing the effects of fertilization treatment and aggregate fractionation on PSB abundance and P-cycling-related gene abundance is of great importance to guide PSB isolation and rational fertilization in agro-ecosystems. For this reason, we collected soils under five fertilization treatments and sieved water-stable soil aggregates (macroaggregate, 250 to 2000 μm; microaggregate, 53 to 250 μm; silt+clay, <53 μm) (8). In this study, we aimed to (i) investigate the numbers of PSB and abundances of P-cycling-related genes (i.e., *gcd* and *bpp*), (ii) estimate abiotic (e.g., soil physicochemical factors) and biotic (e.g., *gcd* and *bpp* abundances) factors on numbers of PSB, and (iii) decipher the performance of PSB for promoting plant growth. Considering that P availability affects the abundances of P-cycling-related genes (6, 7, 29, 30), we hypothesized that P availability would also determine numbers of PSB. Given that P-cycling-related genes can be identified from PSB (10, 17, 31), we also hypothesized that there would be a close linkage between PSB number and P-cycling-related gene abundance. To achieve our purpose and address our hypotheses, we conducted colony plate counting, quantitative PCR, and potted experiments.

## RESULTS

### Numbers of phosphorus-solubilizing bacteria and abundances of P-cycling-related genes in soil aggregates.

**FIG 1:**
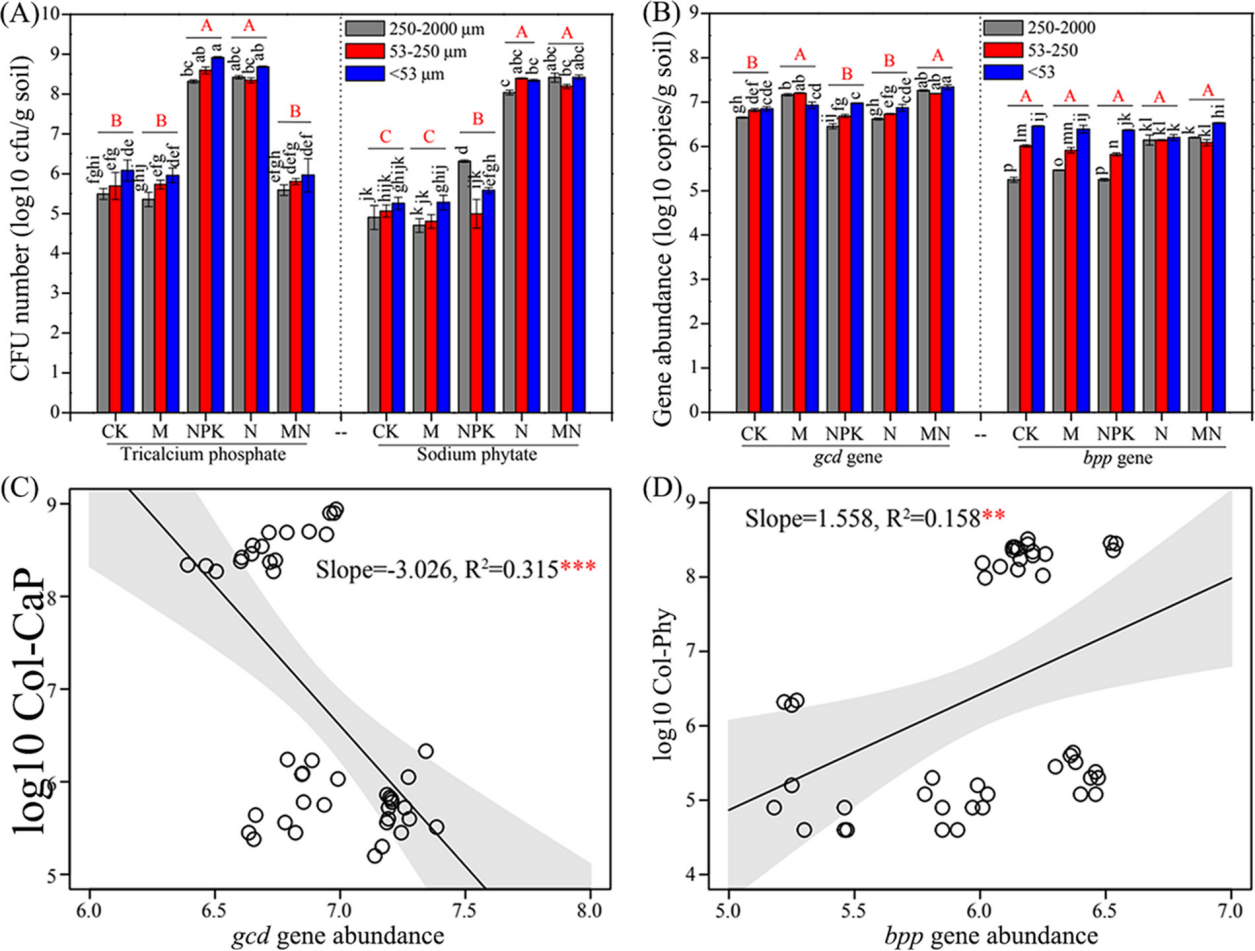
Distribution patterns of phosphate-solubilizing bacteria and P-cycling-related genes and their relationships. (A and B) Numbers of phosphate-solubilizing bacteria (A) and abundances of P-cycling-related genes (B) in different soil aggregates under different fertilization treatments. The lowercase letters above the columns denote significant differences among soil aggregates under five fertilization treatments (*P < *0.05), and capital letters represent significant differences among five fertilization treatments (*P < *0.05). (C and D) Linear regressions reflect relationships between *gcd* gene abundance and tricalcium phosphate-solubilizing bacterial number (Col-CaP) (C) as well as between *bpp* gene abundance and phytate-degrading bacterial number (Col-Phy) (D). Asterisks denote significance (***, *P < *0.001; **, *P < *0.01).

**FIG 2:**
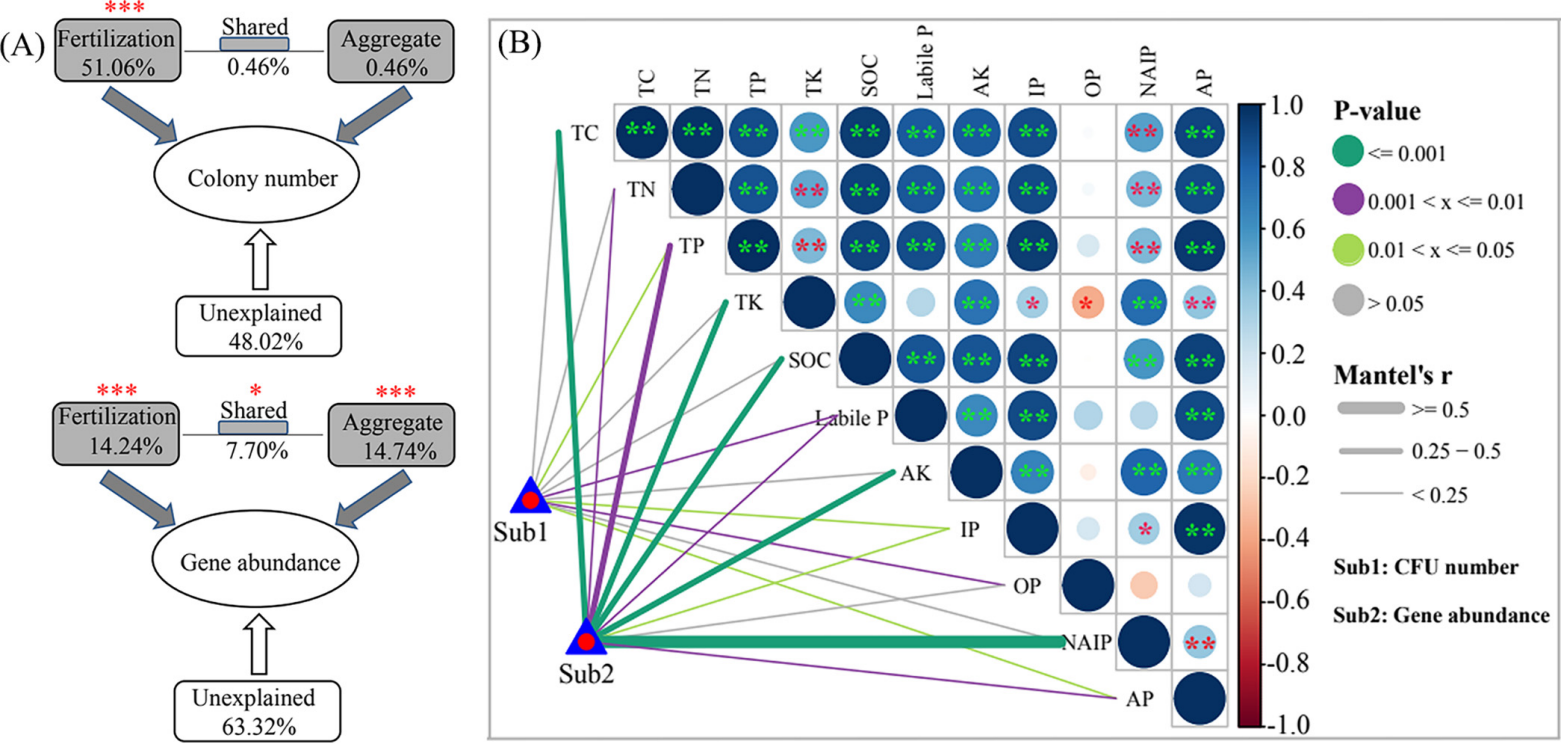
(A and B) PERMANOVA showing effects of fertilization treatment and aggregate fractionation on PSB number and P-cycling-related gene abundance (A) and Mantel’s tests displaying influences of soil physicochemical factors on PSB number and P-cycling-related gene abundance (B). Color gradients and circles denote Spearman’s correlation coefficients. Edge width represents the Mantel’s *r* statistic for the corresponding correlation coefficient, and edge color represents the statistical significance based on 999 permutations. Asterisks denote significance (***, *P < *0.001; **, *P < *0.01; *, *P < *0.05).

The numbers of PSB represented by CFU varied in different soil aggregates (Fig. 1A). The tricalcium phosphate-solubilizing bacterial number (Col-CaP) (1.60 × 10^5^ to 8.77 × 10^8^ CFU/g soil) was significantly higher in NPK (NPK chemical fertilizer) and N (single-nitrogen fertilizer, urea) fertilization treatments than that in CK (control without fertilizer), M (swine manure), and MN (combined swine manure and N fertilizer) fertilization treatments (*P < *0.05). The phytate-degrading bacterial number (Col-Phy) (4.0 × 10^4^ to 3.27 × 10^8^ CFU/g soil) was remarkably higher in N and MN fertilization treatments than that in CK, M, and NPK fertilization treatments (*P < *0.05). In five fertilization treatments, the Col-CaP and Col-Phy were basically slightly higher in silt+clay than that in macroaggregate and microaggregate (*P > *0.05; Fig. 1A). According to permutational multivariate analysis of variance (PERMANOVA) results, fertilization treatment (R^2^ = 51.06%, *P < *0.001) rather than aggregate fractionation (R^2^ = 0.46%, *P > *0.05) showed a significant effect on PSB number (Col-CaP and Col-Phy) (Fig. 2A). Additionally, Col-CaP was significantly higher than Col-Phy in the same soil aggregate with CK, M, and NPK fertilization treatments (*P < *0.05) but the opposite for MN fertilization treatment (*P < *0.05). Col-CaP was slightly higher than Col-Phy in the same soil aggregate with N fertilization treatment (*P > *0.05). These results indicated different distribution patterns of PSB in soil aggregates with different fertilization treatments.

The abundances of P-cycling-related genes varied in different soil aggregates (Fig. 1B). The *gcd* gene (4.32 × 10^6^ to 2.20 × 10^7^ copies/g soil) was more abundant in M and MN fertilization treatments than in other fertilization treatments (*P < *0.05), whereas the *bpp* gene (1.99 × 10^5^ to 3.42 × 10^6^ copies/g soil) showed no significant difference among five fertilization treatments (*P > *0.05). Basically, *gcd* and *bpp* were more abundant in silt+clay than in macroaggregate and microaggregate with the same fertilization treatment (Fig. 1B). Consequently, both fertilization treatment (R^2^ = 14.24%, *P < *0.001) and aggregate fractionation (R^2^ = 14.74%, *P < *0.001) showed significant effects on abundances of P-cycling-related genes (Fig. 2A). In addition, abundances of *gcd*-harboring bacteria were significantly higher than that of *bpp*-harboring bacteria in the same soil aggregate with the same fertilization treatment (*P < *0.01). These results reflected that fertilization treatment and aggregate fractionation might influence the distribution and abundance of *gcd*-harboring bacteria and *bpp*-harboring bacteria.

**FIG 3:**
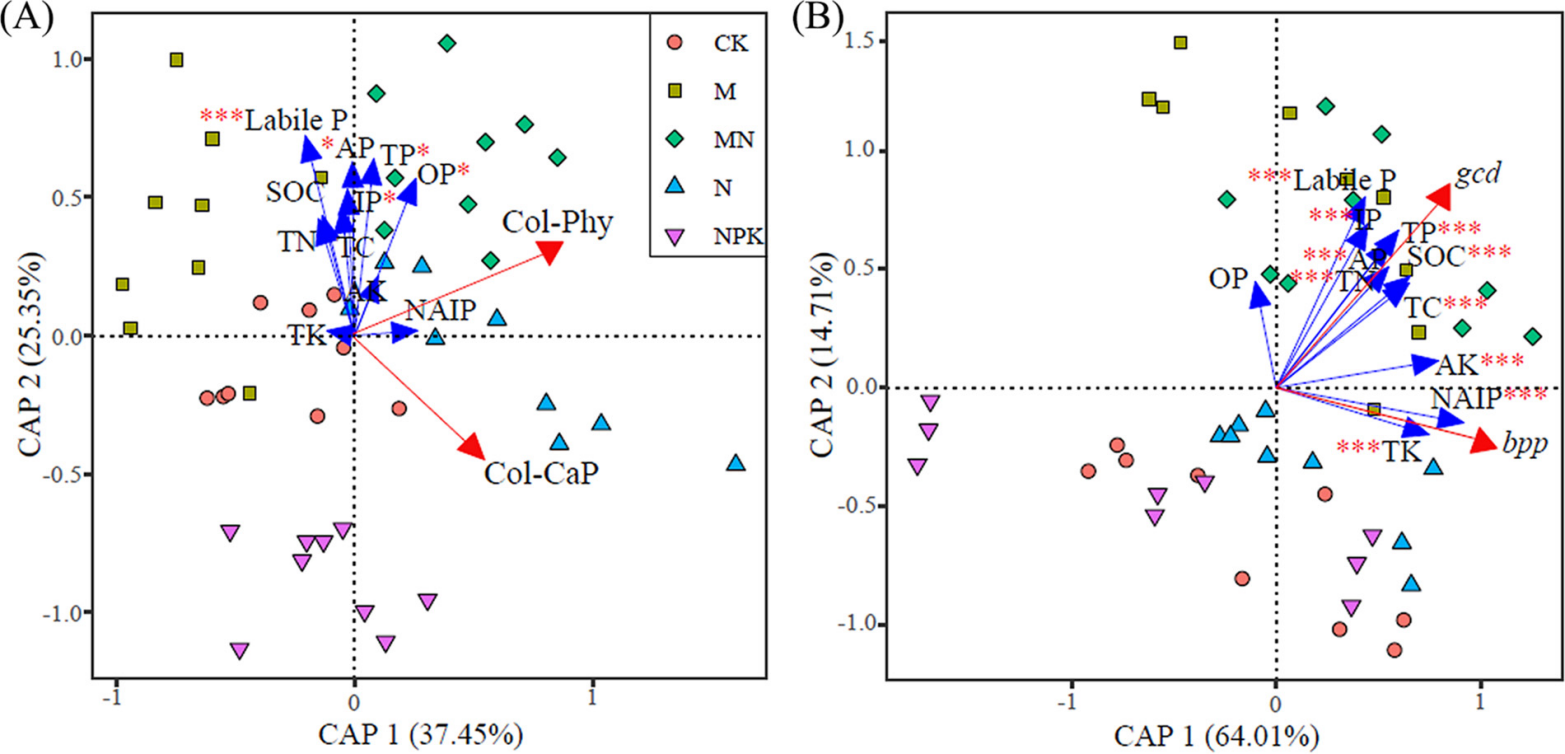
(A and B) Canonical analysis of principal coordinates showing effects of soil physicochemical variables on numbers of PSB (Col-CaP and Col-Phy) (A) and P-cycling-related gene abundance (B). Numbers in parentheses in the axis labels represent the proportion of variance accounted by the principal coordinates. The significance of soil physicochemical factors was determined by applying PERMANOVA and is indicated by asterisks next to the variable names.

Linear regressions reflected that *gcd* gene abundance was significantly negatively correlated with Col-CaP (R^2^ = 0.315, *P < *0.001; Fig. 1C), whereas *bpp* gene abundance was noticeably positively correlated with Col-Phy (R^2^ = 0.158, *P < *0.001; Fig. 1D). Soil nonphosphorus nutrients (i.e., carbon, nitrogen, and potassium) related differently to P fractions (Fig. 2B), and these physicochemical factors showed different correlations with numbers of PSB (i.e., Col-CaP and Col-Phy) and abundances of P-cycling-related genes (i.e., *gcd* and *bpp*) (Table 1). Nonphosphorus nutrients (i.e., total carbon [TC], soil organic carbon [SOC], and total nitrogen [TN]) and P fractions (i.e., total P [TP], labile P, IP, OP, and apatite inorganic P [AP]) were significantly negatively correlated with Col-CaP (*P < *0.05 or *P < *0.01 or *P < *0.001), whereas only OP was significantly correlated with Col-Phy (*P < *0.05). Similarly, nonphosphorus nutrients (i.e., TC, SOC, total nitrogen [TN], total potassium [TK], and available potassium [AK]) and P fractions (i.e., TP, nonapatite inorganic P [NAIP], and AP) were significantly positively correlated with both *gcd* and *bpp* gene abundances (*P < *0.05 or *P < *0.01 or *P < *0.001) (Table 1). According to canonical analysis of principal-coordinate analysis results, soil physicochemical factors explained 62.8% and 78.72% of variations in PSB number and P-cycling-related gene abundance, respectively (Fig. 3). Additionally, P fractions showed significant effects on PSB number and P-cycling-related gene abundance based on PERMANOVA results.

**TABLE 1:** Pearson’s correlations between soil physicochemical factors and phosphate-solubilizing bacterial number and P-cycling-related gene abundance^*a*^

Property	Colony no.		Gene abundance	
	Col-CaP	Col-Phy	*Gcd*	*bpp*
TC	–0.340*	0.088	0.747***	0.451**
SOC	–0.351*	0.017	0.723***	0.524**
TN	–0.348*	0.002	0.755***	0.469**
TP	–0.411**	0.236	0.797***	0.373**
Labile P	–0.576***	0.043	0.784***	0.199
IP	–0.377*	0.123	0.728***	0.258
OP	–0.296*	0.349*	0.019	0.071
NAIP	0.086	0.203	0.489**	0.845***
AP	–0.437**	0.166	0.750***	0.368*
TK	–0.056	–0.083	0.359*	0.748***
AK	–0.111	0.134	0.600***	0.736***

a Abbreviations of physicochemical factors are defined in Materials and Methods. The Col-CaP and Col-Phy denote tricalcium phosphate-solubilizing bacterial number and phytate-degrading bacterial number, respectively. Asterisks denote significance (*, *P *< 0.05; **, *P *< 0.01; ***, *P *< 0.001).

**FIG 4:**
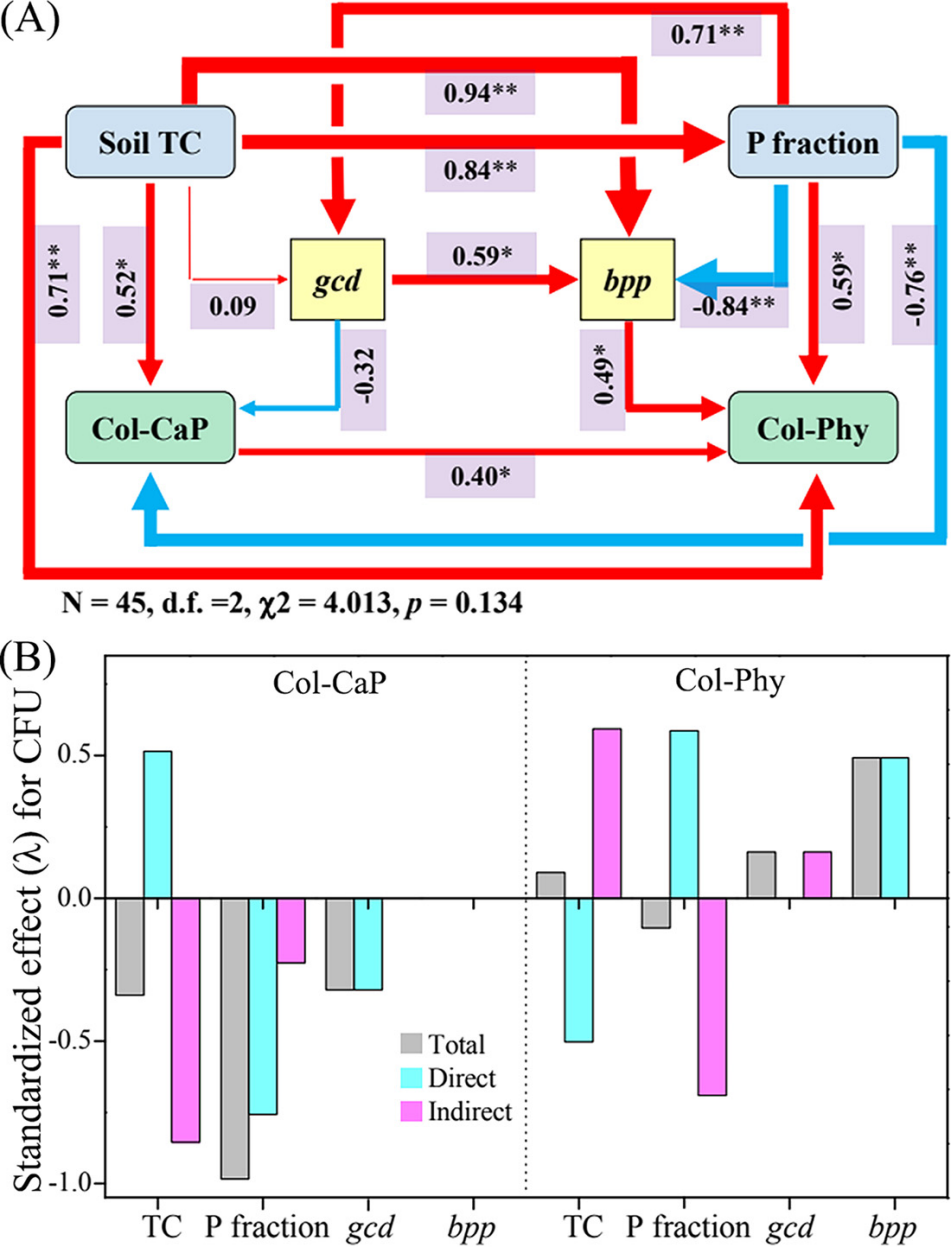
(A) Structural equation model (SEM) showing the hypothesized causal relationships among components, including soil TC, P fractions, *gcd* abundance, *bpp* abundance, Col-CaP, and Col-Phy. The width of the arrows represents the strength of the standardized path coefficient, and values above the lines indicate path coefficients between two parameters. The red and blue lines indicate positive and negative path coefficients, respectively. Asterisks denote significance (**, *P < *0.01; *, *P < *0.05). (B) The direct and indirect effects of abiotic (i.e., soil TC and P fraction) and biotic (i.e., *gcd* and *bpp*) factors on PSB number (i.e., Col-CaP and Col-Phy).

A structural equation model showed close linkage among soil TC, P fractions, *gcd* abundance, *bpp* abundance, Col-CaP, and Col-Phy (Fig. 4A). Soil TC displayed a significant positive effect on P fractions, which exhibited significant positive effects on *gcd* abundance and Col-Phy, as well as noticeable negative influences on *bpp* abundance and Col-CaP. The model displayed a good fit to our data, as indicated by the nonsignificant χ^2^ test (sample number = 45, χ^2^ = 4.013, degree of freedom = 2, *P = *0.134). Additionally, soil TC and P fractions, rather than *gcd* abundance, showed strong indirect and direct effects on Col-CaP, respectively (Fig. 4B). Soil TC and P fractions displayed both strong indirect and direct effects on Col-Phy, whereas *bpp* abundance showed a relatively strong direct effect on Col-Phy. These results indicated that soil TC, P fractions, and gene abundance would affect PSB number.

### P solubilization and plant-growth promoting performance of PSB.

**FIG 5:**
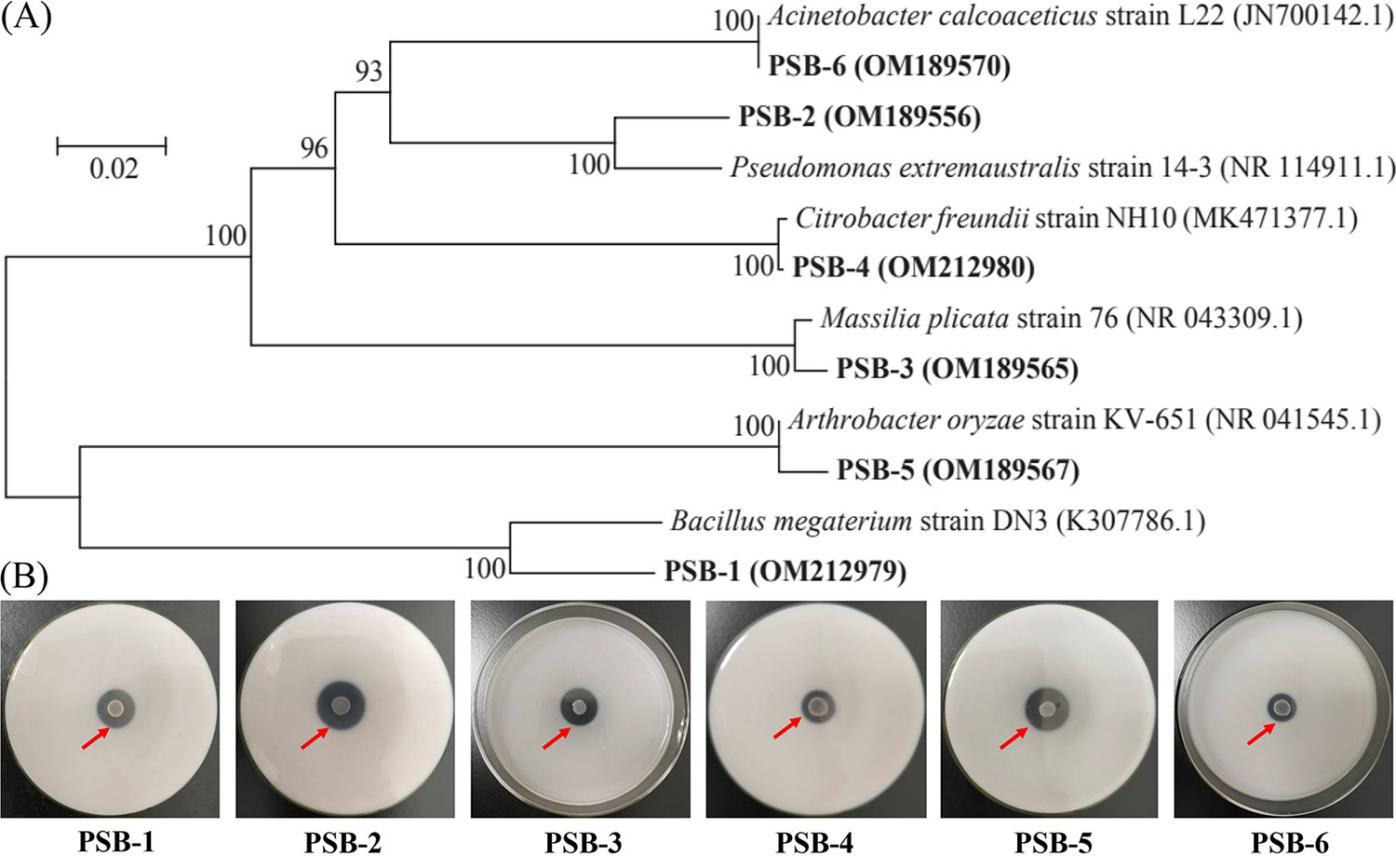
(A and B) Neighbor-joining phylogenetic tree of six PSB based on 16S rRNA gene sequences (A) and halo zones for P-solubilizing performance of six PSB (B).

We isolated tricalcium phosphate-solubilizing bacteria to investigate their P-solubilizing potentials. Isolated PSB (accession numbers OM212979, OM189556, OM189565, OM212980, OM189567, and OM189570) were identified as *Bacillus*, Pseudomonas, *Massilia*, *Citrobacter*, *Arthrobacter*, and Acinetobacter genera according to a phylogenetic tree based on 16S rRNA gene sequences (Fig. 5A). PSB-2 and PSB-5 displayed better performance for utilizing tricalcium phosphate than PSB-1, PSB-3, PSB-4, and PSB-6 based on the halo diameter (HD)/colony diameter (CD) ratio (Fig. 5B).

**FIG 6:**
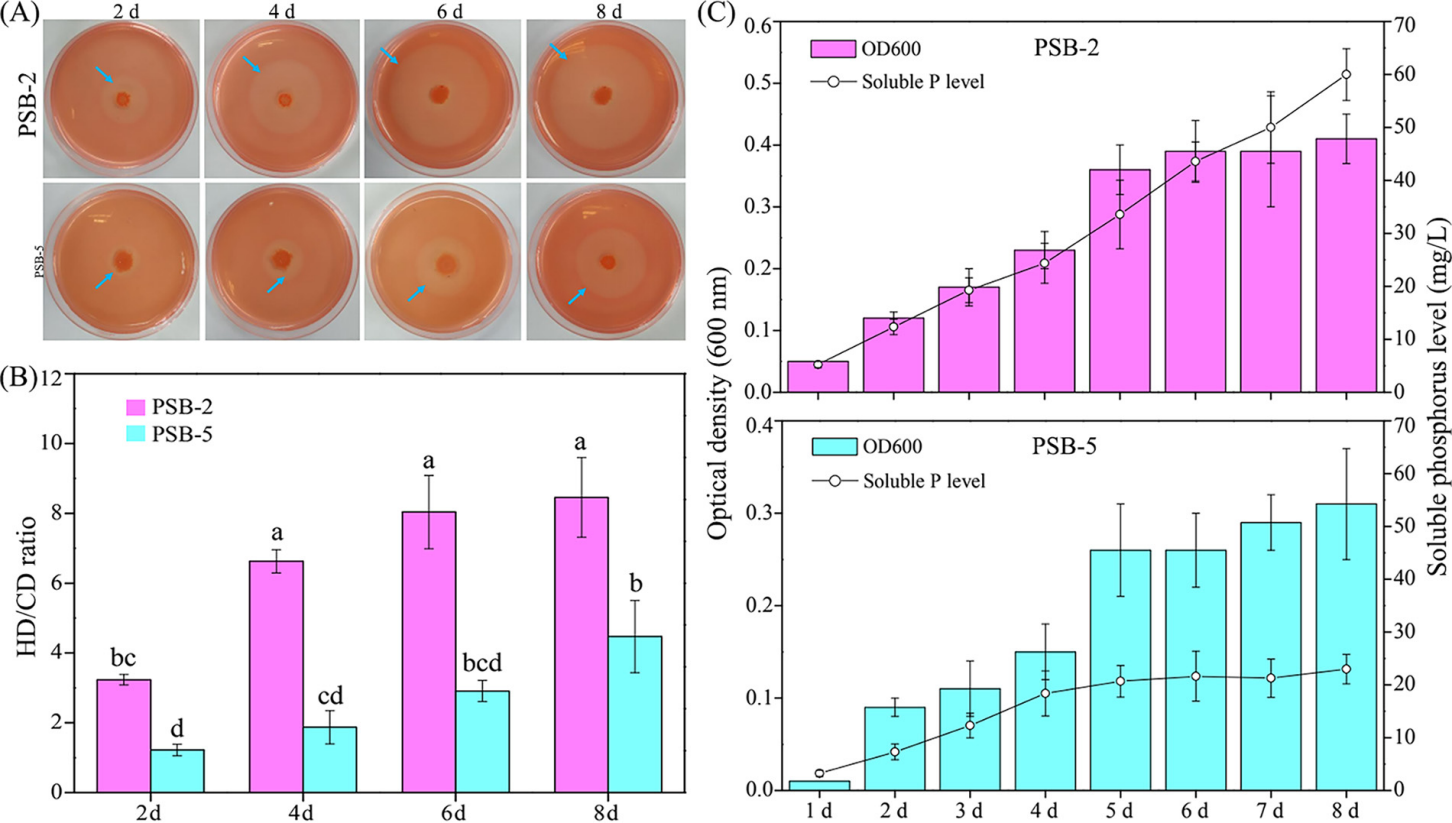
P-solubilizing performance of strains PSB-2 and PSB-5 in both solid and liquid media. (A) Halo zones for P-solubilizing performance of strains PSB-2 and PSB-5 for 8 days. (B) Ratios of halo diameter (HD) to colony diameter (CD) of strains PSB-2 and PSB-5. Lowercase letters above the column denote significance (*P < *0.05). (C) Bacterial density represented by optical density at 600 nm and soluble P levels of strains PSB-2 and PSB-5.

Subsequently, we evaluated the growth characteristics and tricalcium phosphate-solubilizing performances of PSB-2 and PSB-5 (Fig. 6). The HD/CD ratios increased with the incubation time (Fig. 6A and B). The HD/CD ratios for PSB-2 were significantly higher than that for PSB-5 at the same period (Fig. 6B). Cell densities (optical density at 600 nm [OD_600_]) and soluble P levels of PSB-2 cultured in liquid National Botanical Research Institutes phosphate growth medium (NBRIP) media were significantly higher than that for PSB-5 at the same period (*P < *0.05; Fig. 6C). The highest soluble P levels were observed at day 8, showing 59.98 mg/L for PSB-2 and 22.98 mg/L for PSB-5. PSB-2 and PSB-5 experienced significantly fast growth from day 1 to day 5 (*P < *0.05), and growth tended to be stable after day 5 (*P > *0.05). The soluble P levels of PSB-5 displayed a fast increase during 5 days and presented slight fluctuation after day 5. In contrast, soluble P levels of PSB-2 exhibited significantly fast increases during 8 days (*P < *0.05; Fig. 6C).

**FIG 7:**
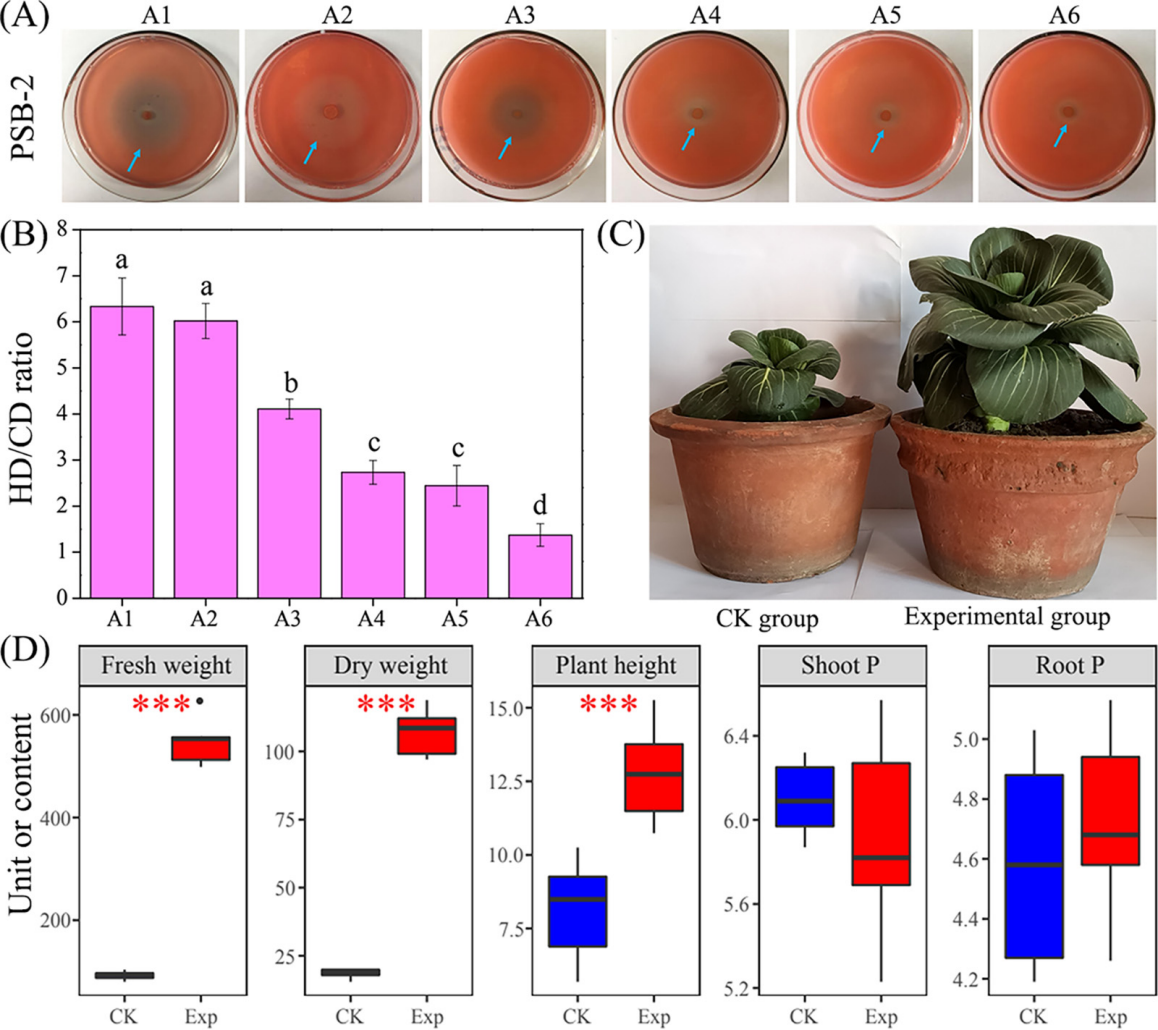
P-solubilizing performance and plant-growth-promoting ability of strain PSB-2. (A) Halo zones of strain PSB-2 in solid NBRIP media containing different amounts of tricalcium phosphate (i.e., A1, A2, A3, A4, A5, and A6). (B) Ratios of halo diameter (HD) to colony diameter (CD) of strain PSB-2 in solid NBRIP media containing different amounts of tricalcium phosphate. Lowercase letters above the column denote significance (*P < *0.05). (C) Plant-growth-promoting performance of strain PSB-2. (D) Differences in vegetation properties (i.e., plant fresh weight, plant dry weight, plant height, shoot P, and root P) between the no PSB-2 addition group (CK) and the PSB-2 addition group (Exp). Units for parameters: g (fresh weight and dry weight), cm (plant height), mg/g dry weight (shoot P and root P). Asterisks denote significance (***, *P < *0.001).

We further investigated the tricalcium phosphate-solubilizing performance and plant-growth-promoting ability of strain PSB-2 (Fig. 7). The HD/CD ratios significantly decreased with the increase in tricalcium phosphate addition amount (*P < *0.05; Fig. 7A and B), suggesting that excessive P addition did not promote P solubilization. The PSB-2 bacterial suspension enhanced the growth of Chinese cabbage (Fig. 7C), showing significantly higher fresh weight, dry weight, and plant height (Fig. 7D). However, the shoot P and root P displayed no significant differences between control group and experimental group. These results indicated that abundant P addition did not enhance tricalcium phosphate solubilization, and PSB-2 could be a candidate for plant-growth-promoting bacteria.

## DISCUSSION

### P fractions affecting P-cycling-related gene abundance and PSB number.

Fertilization treatments showed a greater effect on PSB numbers than did soil aggregates, which is similar to findings in previous studies reporting that the abundance of phosphorus-solubilizing bacteria is closely related to NPK or N fertilization application (32, 33). In addition, both fertilization treatment and aggregate fractionation affected abundances of *bpp* and *gcd* genes, which is similar to a prior finding (34). To our knowledge, this is the first report that N fertilization treatment and silt+clay tend to enrich culturable tricalcium phosphate-solubilizing bacteria and phytate-degrading bacteria. Both inorganic and organic fertilization treatments can alter P fractionation (7, 30, 35–37) and soil aggregate fractionation (8, 35). Additionally, soil aggregate fractionation also shapes P fractionation (8, 35, 38). Therefore, we might conjecture that strong effects of fertilization treatment on both P-cycling-related gene abundance and PSB number might occur via two pathways: (i) directly shaping P fractionation and (ii) indirectly affecting soil aggregate fractionation.

Prior studies have reported that P components (e.g., available P and inorganic P) affect abundances of P-cycling-related genes (e.g., *bpp*, *phoD*, and *gcd*) (6–8, 34). For instance, water-soluble P significantly positively affected abundances of *bpp* and *gcd* genes (10). The abundances of *gcd* and *bpp* genes increased toward higher P levels, which is not in line with findings describing that *gcd* and *bpp* are more abundant under conditions with insufficient P (29, 39, 40). The divergence might be due to differences in phosphorus availability and P-solubilizing potentials of PSBs among different habitats. The elevated P (i.e., TP, labile P, IP, OP, and AP) levels decreased numbers of tricalcium phosphate-solubilizing bacteria, which is consistent with a prior study (41) and differs from an earlier study (42). Additionally, abundant OP increased the number of phytate-degrading bacteria, which is in accordance with the previous finding reporting that more phytate-degrading bacteria can be found in P-sufficient soils than that in P-insufficient soils (14). These results and findings raise the question of why P fractions are so often such good predictors of PSB number and P-cycling-related gene abundance. One possible reason is that P fractions are utilized in order—first, soluble orthophosphate ions in soils and then insoluble inorganic/organic P sources. Previously published literature has reported that inorganic insoluble P can be easily dissolved, depending on the amount of released organic acids (9, 17, 43, 44), whereas phytate is relatively hard to be mineralized by soil PSB (5, 14, 15). In addition, it was worth noticing that carbon and nitrogen levels had certain influences on numbers of PSB and abundances of P-cycling-related genes, suggesting that the utilization of P sources also relies on the levels of carbon and nitrogen sources (45). This phenomenon might be due to strong linkage among carbon, nitrogen, and phosphorus cycles (46).

### P-cycling-related gene abundance indicating PSB number.

A strong positive linkage was found between *bpp* gene abundance and phytate-degrading bacterial number, which is expected, and this might be because phytate is a relatively stable compound (5). Additionally, the *bpp* gene is more conservative and widespread than other phytate-degrading-related genes (e.g., *ptp* and *hap*) (5, 16, 47). A close negative relationship was found between *gcd* gene abundance and tricalcium phosphate-solubilizing bacterial number, which is beyond our expectations, and this phenomenon might be attributed to nutrient level and microbial interaction. Abundant P sources via fertilization treatment meet P demand for microorganisms (6, 30, 34), which in turn weaken and even deprive solubilizing potentials for tricalcium phosphate of phosphate-solubilizing bacteria. Microorganisms can cooperate directly and indirectly to obtain nutrients from environment to survive (48). Organic P-degrading bacteria can provide free P for *gcd*-harboring bacteria, which might also lead to PSB’s a loss of the ability for P solubilization. Additionally, large populations of soil microorganisms potentially affect environmental pH via releasing CO_2_ (49), which facilitates soil P solubilization and, in turn, affects the P-solubilizing potentials of PSB. We found Pseudomonas sp. strain PSB-2 and *Arthrobacter* sp. strain PSB-5 isolated from same soil exhibited different performances for solubilizing tricalcium phosphate. This phenomenon is similar to earlier findings describing different P-solubilizing performances of PSB derived from the same environment (10, 13, 23, 41, 50). Evolutionary history and environmental heterogeneity determine microbial environmental adaptability for utilizing nutrients (e.g., carbon, nitrogen, phosphorus, and sulfate) (51). Consequently, different P-solubilizing abilities might potentially affect linkage between *gcd* gene abundance and phosphate-solubilizing bacterial number. The colony plate counting approach has been used to determine the numbers of phosphate-solubilizing bacteria in many studies (14, 42); to our knowledge, this might be the first report describing strong linkage between PSB number and P-cycling-related gene abundance in soil aggregates. We therefore conjecture that the finding might guide the isolation of PSB from soils.

### PSB enhancing P availability and promoting plant growth.

The isolated Pseudomonas sp. PSB-2 exhibited good performance for P-solubilizing, and the best P-solubilizing level was 59.98 mg/L. The solubilizing capacity for Ca_3_(PO_4_)_2_ by Pseudomonas sp. PSB-2 is higher than that for Pantoea dispersa Cav.cy3 (<50 mg/L) (52) but lower than that for *Burkholderia* sp. strain PSB-69 (1,393 mg/L) (25) and Serratia marcescens RP8 (974 mg/L) (53). PSB in our and other studies are isolated from different environments (e.g., farmland soils, forest soils, and desert soils) (4, 9, 10, 14, 23), and different PSB showing different P-solubilizing performance might be due to divergences in nutrient availability-induced environmental adaptability at both the taxonomic and phylogenetic levels (54). The Pseudomonas sp. PSB-2 displayed good performance for promoting cabbage’s growth via significantly enhancing plant weight and height. This is similar to Burkholderia cepacia ISOP5 for peanut (55), Enterobacter sp. strain RS1 for chick pea (56), Acinetobacter sp. strain Ac-14 for Arabidopsis thaliana (57), and Pseudomonas monteilii PsF84 for geranium (58). The inoculation of PSB can alter community function of soybean rhizosphere bacteria and increase P-cycling-related gene abundance and thus enhance vegetation properties (24). Previous reports have also reported that inorganic phosphate-solubilizing bacteria can release oxalic, lactic, malic, citric, succinic, and indole-3-acetic acid to enrich soluble P levels (9, 23, 53, 57). The *gcd*-harboring bacteria can release gluconic acid via oxidizing gluconate by producing gluconate dehydrogenase (40, 57). Future studies will investigate the growth-promoting performances of Pseudomonas sp. PSB-2 for different plants and decipher dynamic changes in rhizosphere bacterial community and organic acid type potentially produced by PSB-2.

In conclusion, we found distinct distribution patterns of phosphate-solubilizing bacteria and P-cycling-related genes (i.e., *gcd* and *bpp*) in soil aggregates under different fertilization treatments. We found strong linkages between *gcd* gene abundance and tricalcium phosphate-solubilizing bacterial number, as well as between *bpp* gene abundance and phytate-degrading bacterial number. The phosphate-solubilizing bacteria Pseudomonas sp. PSB-2 and *Arthrobacter* sp. PSB-5 showed good performances for inorganic phosphorus solubilization. The phosphate-solubilizing bacterium Pseudomonas sp. PSB-2 could enhance growth of Chinese cabbage, showing significant increases in plant fresh and dry weight as well as plant height. Our findings extend the knowledge of mechanisms for distribution patterns of phosphate-solubilizing bacteria and P-cycling-related genes in soil aggregates and might guide the isolation of phosphate-solubilizing bacteria. Future work will use multiple techniques (e.g., GeoChip and Illumina MiSeq sequencing) to investigate P-cycling-related bacterial abundance and community composition and optimize condition for P solubilization by PSB and decipher mechanisms for P solubilization at both the gene and protein levels.

## MATERIALS AND METHODS

### Soil collection and physicochemical property determination.

Experimental soil samples were collected from Laiyang Experimental Station in Shandong Province, China, which has applied a summer maize (Zea mays L.) and winter wheat (Triticum aestivum L.) rotation since it was built in 1978. Five fertilization treatments were used, including CK, N, NPK, M, and MN (8). Each fertilization treatment had three replicated plots, and soils were sampled in 2017 after winter wheat harvest (8). We fractionated three water-stable aggregates (i.e., macroaggregate, microaggregate, and silt+clay) by using sieves with different sizes of aperture (8). The obtained soil aggregates were freeze-dried and stored at –80°C for subsequent study. We measured physicochemical properties of soil aggregates previously (8), including TC, TN, TP, TK, SOC, labile P, AK, IP, OP, NAIP, and AP. The detailed descriptions of fertilization treatment, plot design, and physicochemical property determination have been reported previously (8).

### Measurement of gene abundance and PSB number.

We estimated abundances of P-mineralizing-related genes (i.e., *bpp* and *gcd*) for soil aggregates by using quantitative PCR (qPCR). Primer bppF (5′-GAC GCA GCC GAY GAY CCN GCN NTN TGG-3′) and primer bppR (5′-CAG GSC GCA NRT CAN CRT TRT T-3′) were employed to amplify the *bpp* gene (16). Primer gcdF (5′-CGG CGT CAT CCG GGS NTN YRA YRT-3′) and gcdR (5′-GGG CAT GTC CAT GTC CCA NAD RTC RTG-3′) were applied to amplify the *gcd* gene (12). Detailed descriptions of DNA extraction and gene amplification were reported previously (8).

We evaluated the number of PSB using the colony plate counting method (24). Each soil aggregate (1 g) was added to 10 mL of sterile water. The mixture was shaken at 180 rpm for 30 min and then kept standing for 10 min. Based on a stepwise dilution strategy, 0.1 mL of 10^−5^ and 10^−8^ diluents were separately spread on NBRIP containing Ca_3_(PO_4_)_2_ or sodium phytate solid medium and incubated at 30°C for 5 days. The NBRIP contained 10 g/L glucose, 5 g/L Ca_3_(PO_4_)_2_ or sodium phytate, 0.25 g/L MgSO_4_·7H_2_O, 5 g/L MgCl_2_·7H_2_O, 0.2 g/L KCl, 0.1 g/L (NH_4_)_2_SO_4_, 2 mL/L trace element solution, 0.2 g/L cycloheximide, and 18 g/L agar (21). The trace element solution contained 10 g/L EDTA, 2.2 g/L MnSO_4_·H_2_O, 1.0 g/L FeSO_4_·7H_2_O, 0.5 g/L CuSO_4_·5H_2_O, 0.3 g/L CoCl_2_·6H_2_O, 0.2 g/L Na_2_MoO_4_·2H_2_O, and 0.1 g/L CaCl_2_. The initial pH of all media was adjusted to 7.0. After incubation, CFU on each plate were counted, and the number of PSB was calculated according to dilution degree.

### Estimation of tricalcium phosphate solubilization and plant-growth-promoting performance.

Single colonies from the NBRIP containing Ca_3_(PO_4_)_2_ were subcultured by picking and streaking five times to isolate pure colonies. We gained six strains (i.e., PSB-1, PSB-2, PSB-3, PSB-4, PSB-5, and PSB-6) and identified them using simple 16S rRNA gene sequencing at Wuhan Qingke Innovation Biotechnology Co., Ltd. The universal primers 27F (5′-AGA GTT TGA TCC TGG CTC AG-3′) and 1492R (5′-GGT TAC CTT GTT ACG ACT T-3′) were used to amplify the 16S rRNA gene (23). The phylogenetic tree was built using MEGA6 software.

The isolated PSB (i.e., PSB-2 and PSB-5) were incubated in both solid and liquid NBRIP to estimate their solubilization potentials for tricalcium phosphate (10). We inoculated PSB seed cultures (OD = 1.0) to NBRIP and incubated them at 30°C for 8 days. The preparation of seed culture was reported previously (10). We estimated halo diameter and colony diameter on solid BBRIP at 2, 4, 6, and 8 days. As for liquid NBRIP, 1 mL of bacterial suspension was collected each day to determine soluble P concentration and bacterial growth based on the optical density at 600 nm. Additionally, we also inoculated PSB-2 on solid NBRIP containing 1, 2, 5, 6, 8, and 10 mg/L Ca_3_(PO_4_)_2_ and incubated it at 30°C for 3 days.

We assessed the plant-growth-promoting performance of isolated PSB-2 by using potted experiments. The experimental potted soils were collected from an uncultivated field in Wuhan, China (30°28′N, 114°21′E). The original physicochemical properties and processing of experimental potted soils were described previously (24). Two potted treatments were designed: 1,000 g of sieved soil plus 500 mL of sterile water plus 100 NBRIP medium (CK group) and 1,000 g of sieved soil plus 500 mL of sterile water plus 90 mL NBRIP medium plus 10 mL bacterial suspension (PSB incubated at NBRIP for 3 days; 10^7^ CFU/mL) (Exp group). Each treatment had five replicates. Chinese cabbage (Shanghai Qing) seeds were purchased from China National Seed Group, precultivated in sterile nutritious soils, and allowed to grow to about 10-cm length of sprouts (24). Each sprout with the same growth potential was transplanted to each pot as described above, and a bacterial suspension containing PSB-2 was inoculated into cabbage rhizosphere. Each plot was placed in a greenhouse with the cycling treatment of 16-h light and 8-h dark for a total of 20 days. We measured plant fresh weight, plant dry weight, and plant height. We extracted shoot P and root P via H_2_SO_4_-H_2_O_2_ digestion and determined P content using molybdenum-blue colorimetry (24).

### Data analysis.

If not otherwise stated, we estimated significant differences by using the one-way analysis of variance. Permutational multivariate analysis of variance was conducted using the function adonis in the vegan package of R. Canonical analysis of principal coordinates was used to estimate effects of soil physicochemical factors on PSB number and gene abundance using the capscale function in the vegan package. A structural equation model was built to reflect potential linkage among soil physicochemical factors, gene abundance, and PSB number using IBM SPSS Amos v.21. For the structural equation model (SEM), the PC1 value of the first axis of the principal-component analysis, accounting for 99.89% of the total variation, was applied as a proxy for representing P components (i.e., TP, labile P, IP, OP, NAIP, and AP).
